# User perceptions of a point-of-use water filtration device: Qualitative, focus group study

**DOI:** 10.1371/journal.pone.0351861

**Published:** 2026-06-24

**Authors:** Martha Grant Fuller, Josephine N. Najjuma, Frank G. Jacobitz, Keith Macdonald, Gad Ruzaaza Ndaruhutse

**Affiliations:** 1 Hahn School of Nursing and Health Science, University of San Diego, San Diego, California, United States of America; 2 Department of Nursing, Faculty of Medicine, Mbarara University of Science and Technology, Mbarara, Uganda; 3 Department of Mechanical Engineering, Shiley-Marcos School of Engineering, University of San Diego, San Diego, California, United States of America; 4 Department of Biology, College of Arts and Sciences, University of San Diego, San Diego, California, United States of America; 5 Community Based Education Research and Service, Faculty of Medicine, Mbarara University of Science and Technology, Mbarara, Uganda; Cranfield University, UNITED KINGDOM OF GREAT BRITAIN AND NORTHERN IRELAND

## Abstract

Universal access to clean water is sixth of the United Nations sustainable development goals. It has not yet been achieved. Boiling water, for water purification, is cost prohibitive and polluting. Diarrheal diseases linked to unsafe water cause morbidity and mortality in young children in Uganda, where most if the rural population lacks access to treated water. Bacterial water contaminants include *Escherichia coli* and *Salmonella Typhi*. Community perceptions of water quality and mitigation efforts in rural Uganda is unknown. In the absence of safe drinking water, a low-cost method of water purification is needed. In vitro studies of plant xylem filtration found this technique successful in removing coliform bacteria and other pollutants. The purpose of this study was to explore perceptions of water quality impact on the lives and health of residents in a rural community in Southwestern Uganda and obtain reactions to a prototype point-of-use water filtration device to determine the possibility of implementing its use in this community. Qualitative descriptive analysis of translated transcripts of purposefully designed focus groups was conducted. Participants included 36 adult residents of a rural community in Uganda. Groups led by experienced Ugandan facilitators in the local language, no researchers from the USA were present. No participants had access to treated water, past efforts to improve water failed due to lack of follow-up. Community members were aware of the poor quality of their water and described water associated illnesses in family members. Obtaining and boiling water required time, money, and resources with households using an average of 142 liters of water/day. Response to the device was mixed, with some excited at the possibility of using it and others expressing concerns regarding durability and cost. Future efforts must address concerns about costs and durability of any point-of-use device and will require extensive planning for sustainability.

## Introduction

The United Nations (UN) has identified universal access to clean water in the sixth of seventeen sustainable development goals. As of 2024, this goal had not been reached, with only 74% of the world’s population having access to safely managed drinking water [1]. Sub-Saharan Africa has the lowest percentage, only 32% of the population has access to safely managed drinking water. Without access to appropriate water infrastructure, individuals must expend significant amounts of time and energy obtaining and transporting water, leading to loss of ability to pursue education and employment and the risk of injury carrying heavy loads [2]. Worldwide, rural residents are less likely to have access to safely managed drinking water and more likely to use unimproved or surface water for drinking water [1]. In Sub-Saharan Africa, 21.22% of residents only have access to unimproved sources or surface water [1]. Access to clean, safe drinking water is limited in rural Uganda. UN data indicates that 15.84% of the Ugandan population has access to unimproved or surface water sources [1]. Data from the Ugandan National Water and Sewerage Corporation (NWSC) shows provision of treated water to 258 towns, approximately 17% of the Ugandan population [3,4]. Contaminated water is a major public health concern, with exposure to infectious diseases and chemical contaminants causing significant morbidity and mortality [5]. To improve health, public perceptions and understanding of the risks of consumption of contaminated water must be understood [5]. A systematic review by Munene et al found that users of private wells in the United States and Canada were more likely to rely on organoleptic factors rather than water testing to determine if their water was safe, leading to exposure to hazards [6]. A survey of public understanding and perceptions of drinking water quality and health in Southeastern Kenya found that just over half of those surveyed were aware that contaminants could make water unsafe [7]. Residents of Mbarara city in Southwestern Uganda have reported inconsistent access to treated water but have significant concerns about quality and safety of the water [8]. Local resident perception of drinking water quality in rural areas of Southwestern Uganda is unknown, a key starting point for working with communities to develop programs to improve water quality. Point of use (POU) water treatment systems have emerged as a critical decentralized solution for improving water quality at the household level. These interventions are particularly vital in regions where centralized infrastructure is absent, intermittent, or compromised by aging distribution networks. The success of POU water treatment relies on the end user. They must understand the risks associated with their water, identify benefits of the POU device, and be confident in use of the device [9].

### Public health

Diarrheal diseases linked to contaminated drinking water are a leading cause of morbidity and mortality in children under five years of age with 484,000 children dying each year [10]. Almost 60% of the deaths are due to unsafe drinking water and poor sanitation. An analysis of deaths from diarrheal diseases in low-and middle-income countries found the highest risk of death in those in the African region [11]. Most water obtained from sources in southwestern Uganda must be treated to be fit for consumption, many are contaminated with high levels of *Escherichia coli* (*E. coli*) a common bacterial cause of diarrheal disease [12]. This lack of safe drinking water is associated with high rates of diarrheal diseases, one of the top five causes of death in children under age five [13]. A study in Entebbe, Uganda found a 73.5% decrease in incidence of diarrheal disease in children under 5 years of age in families obtaining water from a protected water source [14]. In addition to bacterial contaminants, water in rural Southwestern Uganda has been found to have high levels of iron, fluoride, and lead [15]. Without access to protected water, those in rural areas have no alternative but to use water that is potentially contaminated.

### Household water treatment

Point-of-use (POU) water treatment systems have emerged as a critical decentralized solution for improving household water quality. These interventions are vital where centralized infrastructure is absent, intermittent, or compromised by aging distribution networks. While various pathogens, including protozoa, viruses, and helminths, threaten public health, *E. coli* remains the gold standard indicator for fecal contamination. The World Health Organization (WHO) International Scheme [16] utilizes *E. coli* as the primary bacterial metric for evaluating POU efficacy in laboratory and field settings, requiring a target of 0 CFU per 100 mL for safe water.

Pooi and Ng [17] found that POU systems provide a final barrier against microbial pathogens immediately before consumption, bridging the water quality degradation that occurs between an improved source and the home. This decentralized treatment is essential to prevent negative outcomes from recontamination during transport and storage. Storage remains a frequent failure point where water that was compliant at the source becomes contaminated due to poor hygiene or improper vessel management by the time it reaches the user.

A United-States-based study found that these devices can be cost effective, reducing waterborne illness risks when water is contaminated after treatment [18]. Similarly, a systematic review and meta-analysis of POU water filtration devices found they reduced diarrheal disease risks for children in low- and middle-income countries [19].

POU device performance is quantified by its Log Reduction Value LRV = log_10_(C_influent_/C_effluent_). According to the WHO International Scheme [16], Three Star “comprehensive protection” requires a minimum 3-log reduction for bacteria and 4-log reduction for viruses. Ceramic pot and biosand filters often achieve 2-log to 4-log reductions in laboratory settings. However, a randomized controlled trial in Cambodia by Brown et al. [20] found that real world implementation resulted in a lower 46% reduction in diarrheal disease. This performance gap is often attributed to media pore sizes (0.2 to 2.0 μm) and micro-cracks developing over time. Brown et al. [20] noted the protective effect was most pronounced in households with high initial contamination, showing that health risk reduction is closely tied to baseline water quality.

Despite high technical efficacy, sustained reduction of *E. coli* in the field is often attenuated by socio-economic factors. Montgomery and Elimelech [21] argue that for POU technologies to be sustainable, they must transcend mere affordability. Intervention success is heavily dependent on cultural acceptance and local supply chains for replacement parts. Without these components, even advanced filters are frequently abandoned within months of distribution [21].

A review by Sobsey et al. [22] identified ceramic and biosand filters as having high potential for sustained use because they are easy to operate and provide a visible barrier that builds user trust. They highlight that the primary barrier to safe water is not the filter itself, but the risk of recontamination. Even if a filter is efficient, using a contaminated vessel for storage or improper cleaning can re-introduce *E. coli* levels that negate the health benefits. The technical design of a POU system must account for ease of maintenance and the likelihood of user error during daily operation.

#### Plant xylem filtration.

An emerging area of research focuses on the use of plant xylem as a high efficiency, low-cost filtration membrane. Xylem tissue, particularly the sapwood of gymnosperms, contains pit membranes designed to prevent air embolisms while allowing the passage of water. These membranes possess pore sizes ranging from several nanometers to a few hundred nanometers, making them naturally suited for the mechanical exclusion of *E. coli* and other microbial pathogens. Use of a plant-based filter in a POU device was effective in removing bacteria and toxic metals and could be an affordable method for use in low-and middle- income countries [18,19].

Early proof of concept studies by Boutilier et al. [23] demonstrate that gymnosperm sapwood can achieve >4-log reduction of *E. coli*. Building on this, Ramchander et al. [24] performed a comprehensive engineering characterization of gymnosperm sapwood, identifying that a filter thickness of approximately 6.5 mm is optimal to balance high flow rates with structural integrity. Their work confirmed that these filters maintain performance even after storage for up to two years. Xylem filtration has been found to successfully filter 100% of *E. coli* bacteria [25].

Beyond microbial rejection, the plant structure has a significant effect on toxic metals. As demonstrated by Ibrahim et al. [25], xylem tissue acts as a natural cation exchanger. The presence of negatively charged functional groups within the xylem cell walls allows the filter to adsorb positively charged metal ions. Their research showed that these bio-inspired filters can achieve 70–100 percent removal of toxic heavy metals, including lead and copper, from contaminated water sources.

This dual functionality, mechanical sieving for bacteria and chemical adsorption for metals, suggests that xylem can function as a simple, holistic solution for providing potable water in developing countries. It addresses both the immediate threat of fecal contamination and the long-term health risks associated with heavy metal ingestion [24].

The evolution of point of use (POU) technology reflects a shift toward a multi barrier protection strategy. As Narayan and Tilley et al. [26] argue, POU should be viewed as a flexible and scalable intervention integrated into broader water, sanitation, and solid waste management frameworks. This holistic approach is essential in low- and middle-income countries where fragmented infrastructure often leads to system wide failures.

By aligning decentralized water treatment with improved hygiene and waste services, public health gains are more easily sustained. Montgomery and Elimelech [21] emphasize that the successful integration of these systems depends on local institutional support and community engagement. Ultimately, the transition from standalone filters to integrated service chains allows for a more resilient response to both microbial and emerging chemical threats [21,26].

### User perceptions of POU devices for household water treatment

To be successfully used, the design of any POU system including one using xylem filtration, must be acceptable to and address the concerns of the end users. A lack of knowledge and perception of difficulties were barriers to the use of POU devices to filter well water in the United States [9]. A study in Pakistan aimed to understand behavioral and social factors that impact the decision to use POU water filtration systems [27], finding that a knowledge of the health risks posed by contaminated water was a factor leading to use of filtration while associated costs inhibited use. A randomized control trial in Bangladesh by Luoto et al [28], compared four different POU water treatment options in a poor urban population, found low use of all options, even among those with a high concern for diarrheal illness. Use of the filtration device was the highest of all options, but only 29% reported using it, with the time required being a reason for disuse by 27%.

Several studies have explored POU devices in Kenya. A qualitative study in rural Kenya [29] provided both individual and household filters along with an educational public health campaign. Interviews were conducted two months after the campaign. Researchers found that most respondents were aware of contaminated water as a cause of diarrheal disease and had positive responses to use of filtration devices that were provided to them, with disadvantages including inability to repair a broken filter and slow water flow in the individual filters. Studying two different types of filtration devices during a drought in another rural community in Kenya, Wainaina and colleagues [30] found differences in factors associated with consistent use based on the POU device. Ease of obtaining replacement parts, ease of device assembly and the size of the filtration device (could it be used in their home) were important factors influencing use as was approval of people close to the respondents.

Moropeng & Momba [31] carried out a study in a rural South African community and found that households initially accepted the POU devices provided but over time use declined with complaints regarding broken equipment that they could not afford to replace, slow filtration, and a change in the taste of the water. The authors stress the need for community involvement and acceptance of any POU water treatment system.

A qualitative focus group study in rural Ethiopia by Tamene, explored a variety of factors associated with POU water treatment [32]. Many respondents did not treat water because they were unaware of the health risks of water-borne illnesses, others complained about the costs of water treatment, or the length of time required for a gravity water filter to provide water. Almost all participants expressed a desire to have drinking water that tasted good. The need to involve a community in designing and implementing POU water treatment systems using locally available materials are proposed as mechanisms to encourage long term use.

Ogunyoku and colleagues [33] explored community acceptance and use of clay filtration and biosand filters in a rural community in central Uganda. This project included initial community assessment which determined that water treatment was not used often, less than a third of the population routinely boiled water. Physical filtration using a clay pot was preferred by the community but there were concerns about cost and durability.

The perceptions of potential users in rural Uganda to the use and maintenance of a POU plant-based filtration device is not known and is a key step in completing development of these devices.

### The purpose of the study

The aims of this study were to understand the perceptions of local community members, who are potential users of a prototype POU water filter device regarding their water; the quality of the water, efforts required to obtain and purify the water for use, and to hear their responses to a prototype POU water purification device.

## Methods

### Design

This was a qualitative, focus group study. Each focus group was small, to facilitate greater participation from each participant [34–36]. The study took place between April and May 2024. This study was designed and reported in accordance with the Standards for Reporting Qualitative Research (SRQR) guidelines [37].

### Study setting

Kashongi is a Sub County found in Kiruhura District in Southwestern Uganda. This district is in the Rwizi River catchment area, it is a hilly area with some plains, the elevation is between 1200–2000 meters. The steep slopes are associated with soil erosion. There are two rainy seasons (March-May and September-November) with an average precipitation of 1,250 ml, high temperatures range between 18–25°C [38]. It is made up of 66 villages and has an approximated population of about 37,000 people. People occupying Kashongi are majorly Banyankore, one of the major ethnic groups in Uganda. The main economic activity in this area is farming, primarily cattle keeping. There is little or no access to tap water in Kashongi Sub County and there is no access to treated water from the Ugandan NWSC.

**Fig 1:**
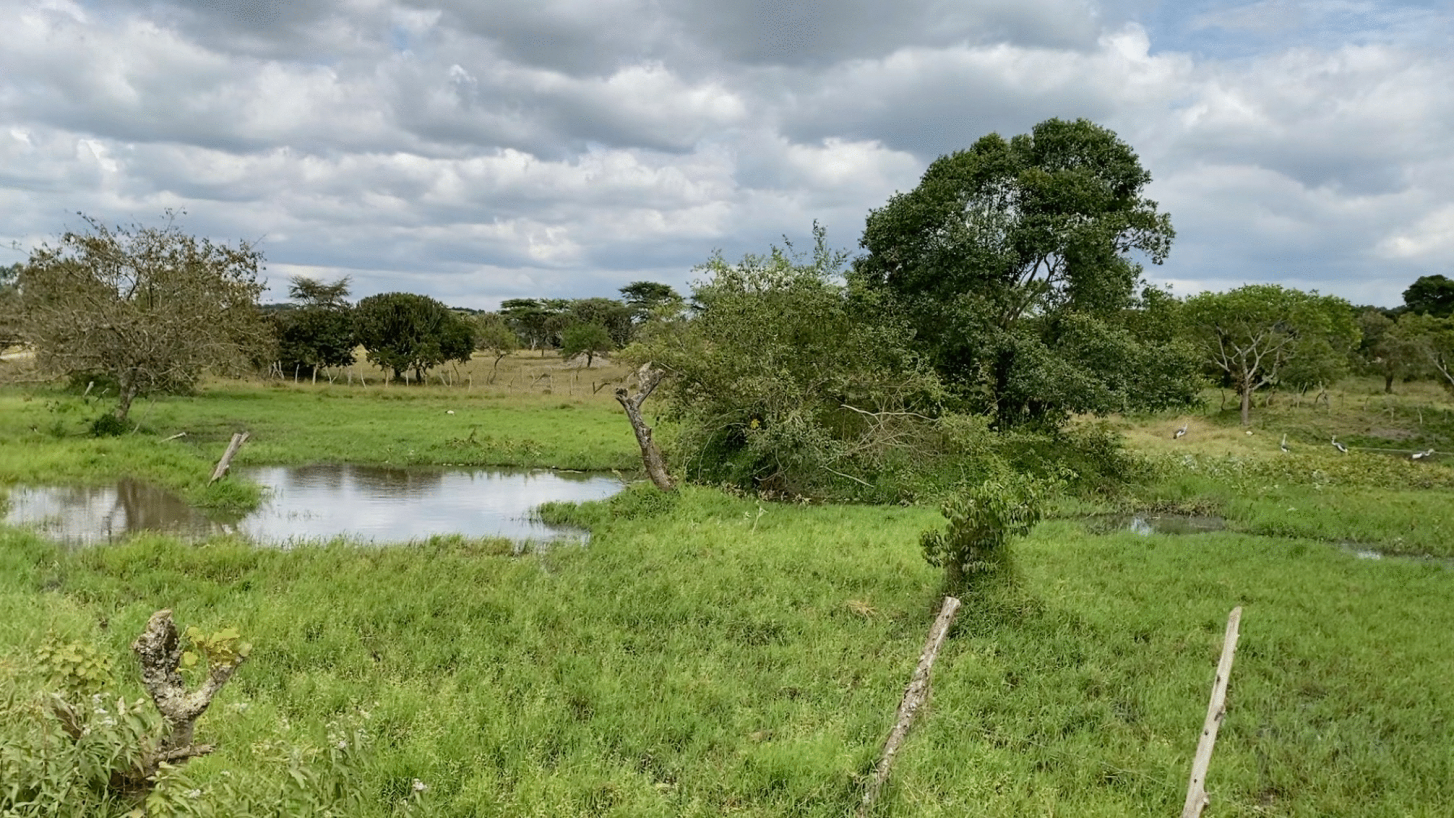
Large dam.

**Fig 2:**
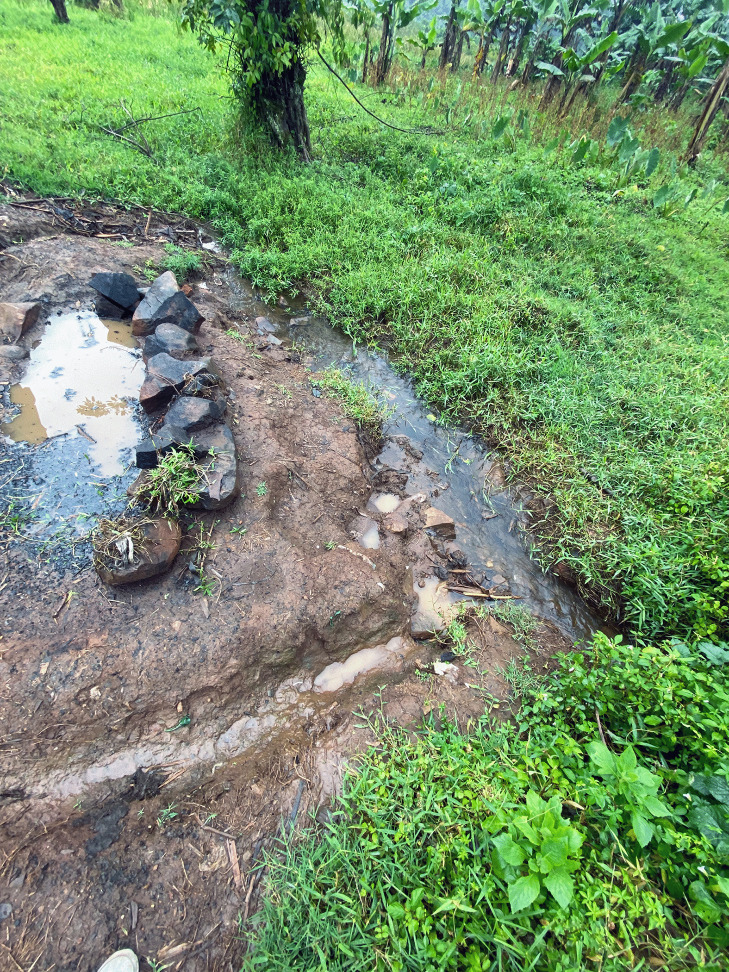
Small dam.

Community water supply is primarily supplied by two dams, the Kyenshama and Mizi dams. Both dams were constructed by the government. There are other small dams owned and controlled by the individuals and small groups that constructed them on their farms. Figs 1 and 2 show examples of these dams. Some individuals in Kashongi use rainwater for home use. The dams are the primary water supply for domestic use and for cattle. The average income of this community is less than $2.00/person/day.

### Point of use device

**Fig 3:**
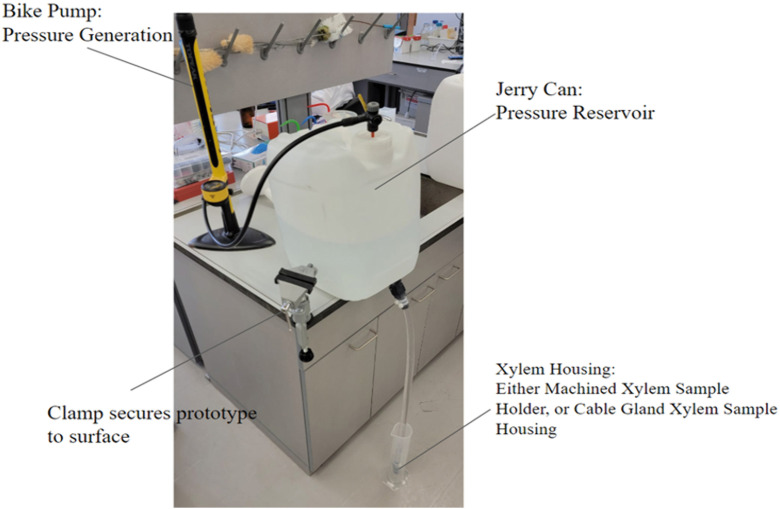
Prototype point of use water device with xylem filtration.

This project utilized a prototype POU xylem filtration device that is currently under development in the Shiley-Marcos School of Engineering at the University of San Diego (Fig 3). The plant used as a xylem source was *pinus halepensis* (Aleppo or Jerusalem Pine, locally known as “pine”). This device uses a bicycle pump to push water through a plant-based filter housed in the xylem housing. The supplies and equipment needed are relatively inexpensive (a total of approximately $11 US Dollars or just under 40,000 Uganda Schillings) and readily available, while the filtration matrix (plant) is renewable.

### Participants

Participants were identified by the contact Village Leader, who told them about the study and made an appointment with them to join the focus group. All participants who were invited to participate in the study, joined voluntarily. The focus groups were small, six participants each, segregated by age and gender to encourage more open conversation based on Ugandan cultural norms [39,40].

### Data collection

The focus group discussions (FGD)were led by an experienced, female trained research assistant speaking in the local language, Runyankore. The FGD leader had a nursing background and was trained on how the device works. There was a note-taker present, and an audio recording was made. The same leader led all the focus group interviews. Demographic information was collected prior to the FGD. The discussion was semi-structured using a focus group guide which was translated to Runyankore. The interviews took an average of 45 minutes. After this, participants were shown the prototype POU device and given a demonstration, and participant perceptions of the device were elicited as part of the FGD. The recordings were transcribed verbatim in Runyankore and then translated to English. The English transcripts were then reviewed by the interviewer for comments and corrections. The interviews took place during the day at a shade (covered waiting area) at a community health center in Kashongi village. Data was collected until saturation was reached. None of the researchers from the United States were present, to avoid any potential power dynamics that might inhibit participants from speaking freely [41]. Likewise, authors from Uganda did not take part in the data collection.

### Discussion guide

The focus group discussion guide/script focused on the areas identified as study goals and had initial prompts focusing on water quality and related issues (S1 Appendix in S1 File). The interview guide was developed by MGF and JNN and reviewed and edited by the team prior to use for data collection. There were two sections, the first addressed general questions about water based on researcher experience and prior research done in East Africa [7]. The second had questions specifically designed to elicit participant response to the prototype POU device.

### Researcher characteristics

Researchers were experienced in conducting qualitative research. The multidisciplinary research team was drawn from two institutions, the Mbarara University of Science and Technology (Uganda) and the University of San Diego (United States) and included nursing and public health professionals, an engineer, and a biologist. None of the researchers had any relationship with any of the participants.

### Data analysis

Data analysis was conducted by four members of the team (two nurses (MGF, JNN), one biologist (KM), and one engineer (FJ) using qualitative description. Qualitative Description is a research technique that aims to represent the experiences and thoughts of participants and not to develop theory [42]. Researchers remain close to the data, provide a description of the phenomenon, and perform data collection in the setting of the participants [43]. This research technique has been proposed for use in studies to plan interventions to address disparities in health, enhancing understanding of the needs of under-resourced populations [44]. Each researcher independently reviewed the English language transcripts using an Excel® template to identify themes and important quotations. This program was available to all researchers and preferable to other available software programs. Units of study included group (themes that arose in each group) and participant (specific comments or ideas from an individual group member). Two researchers (JNN & MGF) reviewed all analysis and identified key topics.

## Results

### Focus groups

**Fig 4:**
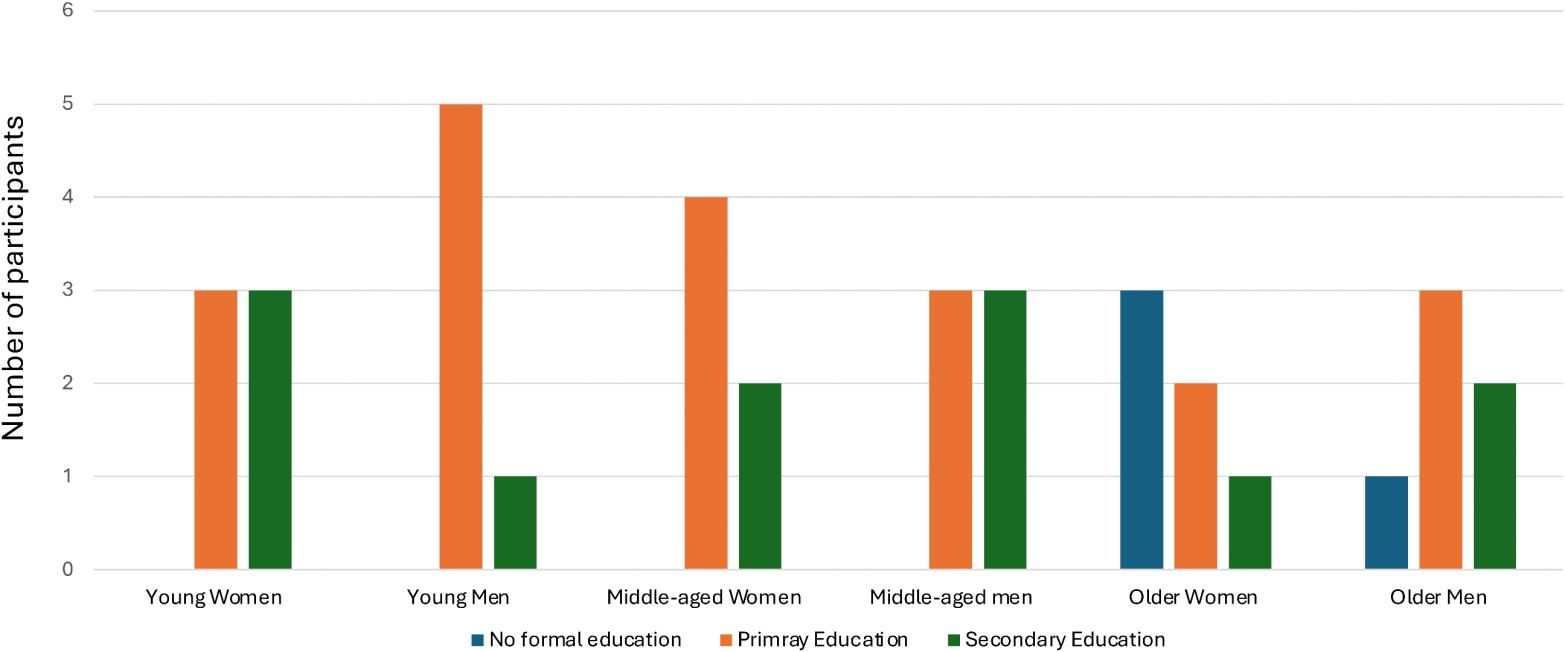
Educational attainment of group members.

There were thirty-six participants, six each in six focus groups was limited by age category and gender. Household size varied as did the quantity of water required. Most described water-related illnesses in their households in the past year (Table 1). Educational levels varied with older participants having the lowest levels of formal education, and no more than 50% of any group having secondary education. No participants had greater than secondary education, see Fig 4.

**Table 1:** Demographics of focus groups.

Group Description N = 6 for each group *M* age in years (range)	Household size, *M* (range)	No. of children < 12 years, per household *M* (range)	No. of water-related illnesses per household *M* (range)	Liters of water per day/household *M* (range)
Young women 20 (18-22)	12 (7-17)	3 (1-5)	1.3 (1‒4)	344 (200‒500)*
Young men 19.5 (18-22)	10 (8-12)	2 (1-3)	**	**
Middle-aged women 38.5 (28–52)	7 (2-11)	2 (1-4)	5.3(2‒12)	78 (40‒120)
Middle-aged men 33 (25–40)	5 (3-7)	3 (1-5)	3.6 (1‒12)	148 (40‒240)
Older women 61.3 (50–74)	5 (2-8)	2 (1-3)	8.5 (0‒24)	72 (60‒80)
Older men 69.5 (64–74)	10 (6-24)	3 (0-18)	1.6 (0‒3)	66.7(40‒80)

* Excludes one using 8,000 L/day for a business.

**missing data.

Each group used the same format and question guide, and had the same interviewer, but there were marked differences in the amount of conversation generated, transcript length ranged from 4 ¾ pages (young men) to 34 pages (older women). There was significant missing data for the group of young men. The group of older women often reinforced and agreed with what was said by other participants and gave longer responses, with rich descriptions. There were common themes across all groups, see Table 2.

**Table 2:** Major themes and subthemes.

Theme	Subthemes
Difficulty obtaining water	Distance Poor availability in dry season Reliance on owners of private wells Hard to transport Safety
Water quality	Poor Dirty Causes illness Animals pollute water
Household water treatment	Time consuming Inadequate Boiling costly Failure of previous efforts
Response to POU device	Decrease reliance on boiling Ease of use for some, not all Slow Costly

### Water sources inconvenient and far

The primary water source for most of the participants (25/36) were sources described interchangeably as “wells” or “dams.” Depending on their location, obtaining water required between one and seven hours. At times, participants asked permission to get water from a private well located nearer to their homes: *“We request a merciful person who owns a farm that will allow us to fetch water.”*

In the dry season water sources were more limited and participants were unable to use the private wells. They described traveling *“a far distance”* (several miles in some cases) to get water from alternative sources including a swamp or river. The dry season also sometimes necessitated hiring someone to transport water due to the distances involved.

### Water quality: Poor

Participants consistently described the water as dirty or contaminated, noting the need for clean water: *“…we are making noise, help us, give us clean water.”*

*“But for us what we see outside we just stay with grid, when we hear that the other place has tap water for drinking, then that for you just fence this well and keep using that for drinking, you just get confused, so what can we do? Are we outside Uganda, or? For us in Rwenjubu Central when shall we get tap water? It’s a question that remains in our hearts; we keep yearning for water.”*

There were multiple descriptions of water related illnesses in their households. Frequency of illnesses ranged from weekly for one participant to one or two times in a year for others. Children were most likely to become ill. *“…people are suffering from typhoid because of the water.” “It is this unclean water that makes us suffer from stomachache. More especially, children are the ones that use it more than older people, which makes them suffer from stomachache.”*

There was no positive description of the water quality. Efforts to clean the water were often unsuccessful. *“…dirty, slime on top even if boiled.”*

Group members described sources of contamination including dirt and animals. Many described more contamination from children and animals. *“…mud water.”*

*“The standard of our water is dirty, because where we fetch it, we share it with livestock like cows, goats. It’s where the children bathe from, when it rains, every dirt is collected.” “Children put their stool in the farm and even an old person like me who is not shy I also help myself in the farm, when it rains it takes all that to the well.”*

Speakers described tadpoles in the water:

*“The standard of our water is not good …after filtering, you find these things I don’t know how they are called, but like small fish, you know it?” Moderator: “Small fish.”*

*Speakers: “(Laughed) when they grow old, they become toads.”*

### Extensive time and effort required for obtaining and preparing water for use

Fetching water and preparing it for use required time and resources. Those who were unable to carry the water themselves needed to pay between 500‒2000 Uganda Schillings for someone to do so. Women bore the primary responsibility for ensuring that their households had adequate water, often sending children to bring water, which brought about anxiety:

*“It is a challenge to send children in the evening … you send a child at 8‒9 pm when it is already late. And then you start lamenting to God about the child’s whereabouts. With all the scares on the way, you start screaming out. These are challenges, sometimes even the wells are bad and some children drown.”*

Preparing water for use required multiple steps: filtration, boiling (twice), removing scum while cooking, cleaning storage containers, and allowing the water to cool. Shortages of and the high cost of firewood and charcoal were barriers to boiling water.

Participants described spending multiple hours for the process ranging from 2 hours to several days. In many cases, it was not easy to differentiate the time spent fetching water from the time required to purify it.

### Household water treatment, failures in past efforts

Participants spontaneously proposed general thoughts on how to improve water quality, including:

*“…like water from the government dams, to make it clean is to put there rules like fencing it so that the livestock does not step in the water, stop people from bathing and even washing their motorcycles, or fencing the wells so that animals could not get to them.”*

Several participants described a variety of past efforts to improve the water quality that were brought to the community by a variety of persons from outside. This has included politicians: *“…there came a Honorable who was called [name redacted] who drained water from there in Buhweju. After it had reached, he was defeated. Since then, the water stopped there, and even electricity.”*

Some efforts were brought by others from outside the community and participants described a lack of follow-up.

*“However, there are some kind people that had taught us to put sand in containers but we also failed to do it. Sometimes they would come and teach us although at times they would tell us to collect sand so that they come to teach us and they don’t turn up.* ***We have never seen them again*** *[emphasis added]. However they taught that idea to a few of us.” “Only that we had got a program, that was brought by Whites***.** *They told us to make pots, they got their cement, they made these….. things for filtering water. They would put stones after putting stones, you put a jerrycan of dirty water in those pots, by the time you try to get it, you find its clean water. But after some time, things didn’t go well. That program stopped.” “In the year 2014, they brought for us some devices for filtering water but a cup could take like an hour. Then later people stopped using them.”*

### Introduction to the POU water filtration device: Group differences in facilitator presentation

Transcripts revealed differences in the verbiage and mode used for introducing the POU device. For most groups, the language focused on the need to remove invisible contamination from the water: *“…helps us to remove dirt from the water which is unseen with our eyes.”*

The group leader(s) went on to note that the device is not yet ready for use but needs the input of potential users, noting that there were no right or wrong responses.

The introduction given to group three (old women) had a lengthy discussion of the mechanics of the device. Several transcripts include the erroneous description of the xylem filter as being eucalyptus wood. The transcript for group six (young women) starts after the introduction was given and the introduction was not captured.

### Perceptions of POU water filtration device

After introduction to the device and a demonstration of how the device worked, participants provided feedback. Respondents had mixed views, both positive: *“…this thing is good for our home use.” “We’ve liked the machine, but I don’t know how we can get it.”*

and negative: *“…takes a lot of time and work for pressure, need to wash it.”*

Positive statements included a hope that they would no longer need to boil water, and that this would reduce the water related illnesses suffered by their children. Some participants felt that this device was simple to use but others expressed concerns that it might be too difficult for some in the community: *“I don’t know whether all of us can manage it.”*

A consistent observation was that the device filtered water very slowly. Some felt that they could do other work while the water was filtering: *“…put pressure and go to do other work,”*

but others felt that it might take away from time needed for other duties: *“…takes time, may not have time to cook.”*

Many expressed concerns regarding cost of the device and how to obtain replacement parts when needed. *“…when you want to buy it, how much? They can ask a lot of money.” “It is expensive, how can it be replaced?”*

Multiple participants were concerned that the device was fragile and might easily be broken by children. One observed that if excess pressure was applied the device might break. There was some misunderstanding about the type of wood necessary for the device: *“And another thing, I’ve seen something that brings out water is not difficult. You just go to the eucalyptus tree and get a stick you burn it in the firewood stove and then use it.”*

## Discussion

All participants were aware that the water available to them was unclean, and there was a good understanding that the water was polluted by human and animal excrement, and that water was the source of illnesses. This differs from a study in Kenya in which half of those studied were unaware of the contaminants in the water [7]. Participants were unhappy with the situation and aware that people living in other communities in Uganda have access to treated tap water.

In agreement with previous findings, residents of Kashongi expend extensive time and effort obtaining water and preparing it for use. Women and children were primarily responsible for this, consistent with previous research [45]. Venkataramanan and colleagues [2] described fractures and other injuries associated with fetching water, however the main concern expressed by the respondents in this study was the risk of drowning for children.

The use of firewood or charcoal to boil water has significant costs, both financial and environmental. It is not known if participants boiled the water indoors or out. Indoor air pollution due to use of these fuels is associated with significant morbidity and mortality in low-and middle-income countries. A systematic review and meta-analysis found a pooled relative risk of 1.25 for mortality in children under 5 years old, with most of the deaths occurring in Africa [46]. In the absence of access to treated water, development of alternative methods for water purification is increasingly important.

The prototype POU device had positives and negatives. Concerns were raised about the durability of the device, cost of the device, and costs to replace it. At cost of approximately $11 US Dollars the device is inexpensive from the perspective of the developers in the United States, but a significant expense for community members. Cost has been a factor leading to users discontinuing use of other POU devices [31–33].

Group members found the device to be slow to use. Participants referenced previous efforts to filter water that took too long therefore community members did not continue to use the device. Previous studies have found that users do not continue to use POU devices for water filtration when the process is time consuming [28,29,31,32]. The designers of the device weighed the options of developing a device operating solely by gravity a slower process, or pressurization, requiring ongoing work to sustain the operation of the device combined with faster filtration. The information from the FGD suggests that community members prefer both ease of use and filtration in a short time, which is likely difficult to achieve with a device based on xylem filtration, even with the use of a pump for pressurization.

There were misunderstandings about the type of wood xylem that will filter the water appropriately. Eucalyptus is widely available but does not filter out coliform bacteria. If this device is revised and made available for wide use, careful education on types of wood to use will be extremely important.

Researchers were unaware of the history of previous attempts to help the community improve their water. Based on participant report, all previous efforts failed, when those initiating the project were no longer present. Any sustainable effort to improve water quality for residents of this community will require equitable involvement and ultimate responsibility with the community. Developing relationships with local communities requires learning their understanding and concerns, a key step in partnering to improve public health. Community-based, participatory action research can be used to partner with local communities to improve public health [47]. Calderón-Villarreal and colleagues [48] used this model to improve water quality for an indigenous community in Mexico. An analysis of studies from 29 different low- and middle-income countries found that community involvement and programs that seek to address the needs of local communities are helpful in successful interventions to improve water and sanitation [49], however this analysis included only one study from Uganda, focused on sanitation.

Supplies required for any POU water treatment device must be affordable and easily available. It is unknown if previous efforts in this area failed because community members could no longer access supplies. The Water and Sanitation Program of the World Bank [50] provides guidance on developing a supply chain, describing an iterative process with interactions between all stakeholders in the context of adequate demand from end users. To be successful, any POU device must be acceptable, affordable, and available in a timely fashion. Developing demand includes consumer (user) education and engagement. Local manufacture of a device may improve profitability and improve the likelihood of sustainability.

Until treated water can be piped into the community, access to clean water will require multiple steps, POU water treatment and improvement of protections to existing water sources. Multiple respondents noted water source contamination from animals. In the city of Mbarara, the largest city near to this rural community, residents have attempted to keep animals away from water sources to prevent this contamination [51]. In this mainly agricultural area, community members, policy makers and other stakeholders need to be involved to separate livestock from the human water sources.

### Limitations

This study was limited to one community/village and may not apply to other communities even in Uganda. Members of this community face water insecurity, but this was not measured. Addition of a validated instrument such as the Household Water InSecurity Experiences Scale [52] could have allowed better understanding of this topic.

In developing the focus group guide, researchers had hoped to differentiate the time required to carry/fetch water from the time required to prepare it for use. The focus group transcriptions were unclear for several groups, and it was not possible to separately quantify the time spent in each activity. This is important since a POU device may change the mechanism and time spent in readying water for use but will not impact the time and costs spent fetching water.

This study involved demonstration of a prototype POU water purification device. This device has been field tested on water samples but has not been tested in vivo, used by residents in daily life.

## Conclusions

This study adds to the literature, revealing the understanding of members of a rural community in Uganda regarding their water quality and the association with illnesses. The community members desire a way to have clean water for themselves and their families. There was interest in and mixed responses to the prototype POU water filtration device that was presented to them, giving important feedback to the engineering team.

Next steps will include further refinements of the device or identifying other methods of purifying water. Ongoing work is being done with faculty-led student engineering teams to identify other plant material that may be used for water filtration. Developing ongoing relationships with key members of the community to develop future research questions and identify sustainable mechanisms to improve water quality for this community. More research needs to be done to quantify the extent to which the community is affected by the water shortages, and time involved in collecting, preparing and using different quantities of water. Testing the device in vivo with different potential users would give more feedback to the engineering team to improve the product.

## Supporting information

S1 File Discussion guide. (PDF)
