# Supplementary material for: User perceptions of a point-of-use water filtration device: Qualitative, focus group study
Source: PLoS One. 2026 Jun 24;21(6):e0351861. doi: 10.1371/journal.pone.0351861 (PMC13293395; doi:10.1371/journal.pone.0351861)
Supplement: S1 File — (PDF) [file pone.0351861.s001.pdf]

Appendix 1
Focus Group Discussion Guide

English:
This was translated by local bilingual research staff into Runyankore. Translation will be culturally appropriate.

Greetings and obtain written consent.

Introduction to the focus group meeting:
"Thank you for coming today. We are interested in hearing from you about how you access water for drinking and cooking and if you have any problems with your drinking water. There are no right or wrong answers. We are recording and writing down your answers so that we may learn what you think. We will not use your names to protect your confidentiality. If your name is used we will de-identify it."

Questions about water:

1) Tell me how you get water?
   a. Where do you get the water you use for drinking and cooking?
   b. Is it the same place all year? (if not, why do you change sources?)
   c. What do you think about the quality of your water? (Do you think it is safe and clean)
   d. If not: is there anything you do to make it safe? (Probe for boiling, decanting and if there are any other methods.)
   e. How much time and resources do you use to make your water safe?
2) In your family who is responsible for getting the water?
   a. How much time does it take?
3) How do you use water in your home?
   a. How much water do you need every day? (Probe for how many jerry cans.)
   b. What activities do you use the water for? (Probe for the specific uses.)
4) How often in the past year have you or any of the people in your household who had diarrhea or other tummy upsets?
   a. What do you think caused the illness?

Introduction to the device:
Sometimes water is dirty (contaminated) with invisible germs that can cause illnesses like diarrhea or chemicals that can cause problems for health over several years. We are working with a team trying to develop a way for people to clean water in their own homes. This is not yet ready for use. To finish making these for people to use, we need to know what you think. We will demonstrate this and allow you to look at and try the device. We would really like your opinions about this device. There are no right or wrong answers.

Demonstration and possible hands practice with the device. Followed by questions:

1) What do you think of the device?
2) What is good about this?
3) What is bad about this?
4) Is it easy to use?
5) You are all busy with many responsibilities. How much time could you or a family member spend using a device like this for cleaning the water you use for drinking and cooking?
   a. Do you have the capacity to use this device?

(Note to interviewers): please collect demographic data (done individually): Age, Educational Level, Gender, Employment, Household description (numbers in the household: adults and children), source(s) of water, how long have you used it/them?)
